# The use of inhaled antibiotic therapy in the treatment of ventilator-associated pneumonia and tracheobronchitis: a systematic review

**DOI:** 10.1186/s12890-016-0202-8

**Published:** 2016-03-08

**Authors:** Christopher J. Russell, Mark S. Shiroishi, Elizabeth Siantz, Brian W. Wu, Cecilia M. Patino

**Affiliations:** Division of Hospital Medicine, Children’s Hospital Los Angeles, 4650 Sunset Blvd, Mailstop #94, Los Angeles, California 90027 USA; Department of Pediatrics, Keck School of Medicine, University of Southern California, Los Angeles, California USA; Department of Radiology, Keck School of Medicine, University of Southern California, Los Angeles, California USA; MD-PhD Program, Keck School of Medicine, University of Southern California, Los Angeles, Calfornia USA; Department of Preventive Medicine, Keck School of Medicine, University of Southern California, Los Angeles, California USA; School of Social Work, University of Southern California, Los Angeles, California USA

**Keywords:** Antibiotics, Inhaled, Antibiotics, Aerosolized, Ventilator-associated pneumonia, Therapy

## Abstract

**Background:**

Ventilator-associated respiratory infections (tracheobronchitis, pneumonia) contribute significant morbidity and mortality to adults receiving care in intensive care units (ICU). Administration of broad-spectrum intravenous antibiotics, the current standard of care, may have systemic adverse effects. The efficacy of aerosolized antibiotics for treatment of ventilator-associated respiratory infections remains unclear. Our objective was to conduct a systematic review of the efficacy of aerosolized antibiotics in the treatment of ventilator-associated pneumonia (VAP) and tracheobronchitis (VAT), using the Cochrane Collaboration guidelines.

**Methods:**

We conducted a search of three databases (PubMed, Web of Knowledge and the Cochrane Collaboration) for randomized, controlled trials studying the use of nebulized antibiotics in VAP and VAT that measured clinical cure (e.g., change in Clinical Pulmonary Infection Score) as an outcome measurement. We augmented the electronic searches with hand searches of the references for any narrative review articles as well as any article included in the systematic review. Included studies were examined for risk of bias using the Cochrane Handbook’s “Risk of Bias” assessment tool.

**Results:**

Six studies met full inclusion criteria. For the systemic review’s primary outcome (clinical cure), two studies found clinically and statistically significant improvements in measures of VAP cure while four found no statistically significant difference in measurements of cure. No studies found inferiority of aerosolized antibiotics. The included studies had various degrees of biases, particularly in the performance and detection bias domains. Given that outcome measures of clinical cure were not uniform, we were unable to conduct a meta-analysis.

**Conclusions:**

There is insufficient evidence for the use of inhaled antibiotic therapy as primary or adjuvant treatment of VAP or VAT. Additional, better-powered randomized-controlled trials are needed to assess the efficacy of inhaled antibiotic therapy for VAP and VAT.

**Electronic supplementary material:**

The online version of this article (doi:10.1186/s12890-016-0202-8) contains supplementary material, which is available to authorized users.

## Background

Ventilator-associated pneumonia (VAP) is a healthcare-associated infection (HAI) that affects 10–28 % of patients receiving mechanical ventilation in the intensive care unit (ICU) [1, 2]. Between 24 and 76 % of patients with VAP die [1], with the mortality attributable to VAP estimated at approximately 10 % [2]. VAP prevention strategies include keeping the patient’s head of the bed raised at 30–45°, use of chlorohexadine oral care, and minimizing mechanical ventilation days through daily readiness-to-wean trials [2]. Treatment of VAP includes administering broad-spectrum intravenous antibiotics targeted at different bacterial classes (e.g., gram-negative bacteria, anaerobes). Adult infectious disease guidelines published in 2005 recommend that efforts should be made to shorten antibiotic courses for VAPs to limit adverse effects [3]. Antibiotics used to treat these infections may cause systemic morbidity, including acute kidney injury and C. difficile infections. Use of broad-spectrum antibiotic therapy against gram-negative bacterial causes of nosocomial ICU pneumonia have been associated with increased bacterial antibiotic resistance rates and selection for more virulent pathogens [4].

One potential therapeutic intervention for VAP and VAT is aerosolized antibiotics. VAP treatment guidelines published in 2005 do not address the use of inhaled antibiotics in the treatment or prevention of VAP and state that “more data are needed on this type of therapy before determining its value.” [3] Since publication of this guideline, several groups have published clinical trials assessing the use of inhaled antibiotics for the prevention and treatment of VAP and VAT. Aerosolized antibiotics may be efficacious in the treatment of respiratory infections by delivering antibiotics directly to the infection source (e.g., lungs), increasing antibiotic concentration to overcome antimicrobial resistance while limiting systemic absorption and decreasing drug toxicity. Animal models testing aerosolized versus intravenous antibiotic therapy for treatment of Pseudomonas aeruginosa demonstrated decreased bacterial load and increased lung tissue antibiotic concentrations with use of aerosolized antibiotics compared with intravenous antibiotics [5, 6]. However, the role of aerosolized antibiotics in the treatment of VAP and VAT in humans remains unclear. The objective of the current systematic review is to assess, in patients with diagnosed VAP or VAT, the efficacy and safety of using inhaled antibiotics for treatment of VAP or VAT.

## Method

### Search strategy

Our systematic review’s study protocol (available upon request) was developed using the guidelines set forth by the Cochrane Collaboration [7] and the Preferred Reporting Items for Systematic Reviews and Meta-Analyses (PRISMA) [8]. In October 2014, we searched three electronic databases (Pubmed, Cochrane Collaboration, and Web of Knowledge) for randomized controlled trials that examine use of inhaled antibiotic therapy in the treatment of VAP and VAT with the technical support of an expert medical science librarian at the University of Southern California (see Additional file 1 for one search example).

### Inclusion criteria

Included studies met all of the following criteria:

1. Patients: Study population was mechanically ventilated patients diagnosed with VAP or VAT
2. Intervention: Use of inhaled antibiotics for treatment of VAP or VAT compared to OR in addition to intravenous antibiotics
3. Reported outcomes of a randomized controlled trial, and
4. Outcome: Reported on some measurement of clinical cure as an outcome (e.g. clinical pulmonary infection score [CPIS])

Studies were excluded if they were written in a language other than English, included patients on chronic positive-pressure ventilation not hospitalized or studied the use of inhaled antibiotics in patients who are not intubated. Multiple published reports from a single study were treated as a single data point.

### Data extraction and analysis

Two authors (MS, BW) independently reviewed and screened all studies for inclusion using a screening tool to increase reproducibility. All study authors reviewed studies when there were disagreements about study inclusion and a consensus was reached. The following information was extracted and documented from studies that met inclusion criteria: study participant characteristics and setting, description of antibiotics studied including route of administration, doses, frequencies, and duration of treatment, the study period, length of follow up and outcomes such as successful treatment.

The methodological quality of all studies that met inclusion criteria was appraised using the Cochrane Collaboration’s tool for assessing the risk of bias in the reporting of clinical trials [7]. This tool rates the quality of a study’s evidence by examining the potential bias in selection, performance, detection, attrition, and reporting. Two authors (MS, BW) assessed the risk of bias for each study that met inclusion criteria study and any discrepancies were reconciled by consensus among the authors.

## Results

**Fig. 1:**
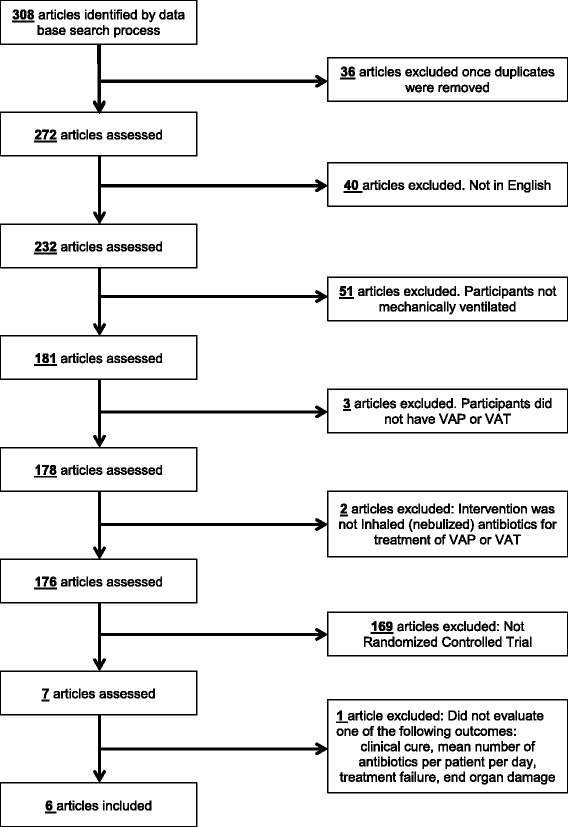
PRISMA diagram

Figure 1 details the study selection process. Our search yielded 272 unduplicated articles, of which 40 were excluded because they were not written in English and did not meet inclusion criteria in review of the English translation. Our review includes six studies describing results of randomized controlled trials on the use of inhaled antibiotics for the treatment of VAP or VAT [9–14]. Three hundred and five patients were enrolled across all studies. One study each tested aerosolized tobramycin [9], amikacin [11], colisthmethate sodium (CMS) [14], and combined amikacin/ceftazidime [10], respectively; two studies based aerosolized antibiotic choice upon culture results at the treating physician’s discretion [12, 13]. Five studies [9, 10, 12–14] utilized clinical measures of VAP treatment success as a primary outcome measure, while one study [11] included measures of treatment success as a secondary outcome. Definition of the main outcome variable, successful treatment, varied widely across studies. Two studies included rigorous and objective measures of cure (e.g., Clinical Pulmonary Infection Score; range 0-12), while others relied on less objective definitions (e.g., Favorable clinical outcome, as defined by the treating physician or study team; Table 1). Third, the study quality and risk for bias varied across the studies (Table 2). Length of the study period varied from 7 to 36 months.

**Table 1:** Study Characteristics, Quality and Results, in Chronological Order

Author and year	Study Participants (age, in years)	Description of intervention	Study period	Length of follow up	Primary Outcome (successful treatment)	Results
Hallal, et al, 2007 [9]	*N* =10	Inhaled tobramycin or IV tobramycin AND IV β-lactam	7 months	28 days	Resolution of VAP	100 % of AA vs. 60 % of IV patients had clinical resolution of VAP. No p value reported.
	Age: 23–72 (Mean age: AA 52.6, IV 53.6)					
Palmer et al, 2008 [12]	*N* = 43	AA or saline placebo AND systemic antibiotics (per treating MD) was given for 14 days or until extubation	12 months	28 days	Centers for Disease Control National Nosocomial Infection Survey diagnosis of ventilator-associated pneumonia (VAP) and clinical pulmonary infection Score (CPIS)	AA group had reduced signs of respiratory infection [Centers for Disease Control National Nosocomial Infection Survey and VAP (73.6 % to 35.7 % vs. placebo: 75 % to 78.6 %) and reduction in clinical pulmonary infection score (−1.42 vs. placebo: + 0.04), (both p ≤ .05).
	Age: 19–92 (Mean age: AA 62.3, placebo 62.7)					
Rattanaumpawan et al, 2010 [14]	*N* = 100 (Mean age: AA 70.2, placebo 66.2)	Nebulized colistimethate sodium or nebulized sterile normal saline AND systemic antibiotics per treating MD	38 months	28 days	Favorable clinical outcome	Favorable clinical outcome was 51.0 % in the AA group and 53.1 % in the placebo group (*p* = 0.84). Significant increase in favorable microbiological outcome in AA vs. placebo group (60.9 vs. 38.2 %; *p* = 0.03)
Lu et al, 2011 [10]	*N* = 40 patients Ages 43–77 (Mean age: AA 58, IV 60)	Nebulized ceftazidime and amikacin OR IV ceftazidime and amikacin/ciprofloxacin.	36 month	28 days	Successful treatment	AA and IV groups performed similar in terms of successful treatment (70 vs. 55 %; *p* = 0.33).
Niederman et al, 2012 [11]	*N* = 69 (Mean age: AA q12h 56.1, AA q24h 62.8, or placebo 62.0)	Inhaled amikacin (BAY41-6551) q12h, q24h, or placebo q12h for 7–14 days, plus standard IV antibiotics	13 months	31 days	Clinical cure (secondary study outcome)	Clinical cure achieved in 93.8 % (AA q12h), 75 % (AA q24h) and 87.5 % (placebo; *p* = 0.467).
Palmer et al, 2014 [13]	*N* = 43 (Mean age AA 57.5, placebo 60.6)	AA or saline placebo AND systemic antibiotics (per treating MD) was given for 14 days or until extubation	Does not state	14 days	Clinical Pulmonary Infection Score (CPIS)	CPIS score in AA significantly reduced when compared to placebo (Mean ± SE AA: 9.3 ± 2.7 to 5.3 ± 2.6 vs. placebo: 8.0 ± 23 to 8.6 ± 2.10; *p* = 0.0008)

*Abbreviations*: *AA* aerosolized antibiotics, *CPIS* Clinical Pulmonary Infection Score; *SEM* standard error of the mean, *VAP* ventilator-associated pneumonia

**Table 2:** Bias Assessment for Individual Studies

Study	Selection Bias	Performance Bias	Detection Bias	Attrition Bias	Reporting Bias	Conflict of Interest
Hallal, et al. (2007) [9]	Low	Low	Low	Low	Low	Unclear
Palmer et al. (2008) [12]	Low	Low	Low	High	Low	High
Rattanaumpawan et al. (2010) [14]	Unclear	High	Unclear	Low	Low	Low
Lu et al. (2011) [10]	Unclear	High	High	Low	Low	Low
Niederman et al. (2012) [11]	Low	High	High	High	High	High
Palmer et al. (2014) [13]	Low	Low	Low	Low	Low	Unclear

Overall, two studies [12, 13] found statistically significant improvements in the primary clinical outcome (clinical cure). Palmer and colleagues [12] reported that aerosolized antibiotics resulted in significantly reduced signs of respiratory infection and clinical pulmonary infection score when compared to placebo score (mean change ± SD = −1.42 ± 2.3; *p* = 0.02). With respect to their secondary outcomes, this study reported that aerosolized antibiotics resulted in lower white blood cell count at day 14 (in 10^3^/mm^3^: AA: 9.2 ± 3.3 vs placebo: 14.9 ± 8.1; *p* = 0.02), reduced bacterial resistance (AA: 0 % vs placebo: 33 %; *p* < 0.01), reduced use of systemic antibiotics (AA: 47 % vs placebo: 70.8 %; *p* < 0.05) and increased ventilator weaning (AA: 80 % vs placebo: 45 %; *p* < 0.05). A second study by Palmer [13] found that compared with placebo, aerosolized antibiotics significantly reduced clinical pulmonary infection score (AA: 9.3 ± 2.7 to 5.3 ± 2.6 vs placebo: 8.0 ± 2.1 to 8.6 ± 2.6; *p* < 0.001).

Three studies [9, 11, 14] found no difference in clinical cure rates when comparing intravenous antibiotics to intravenous antibiotics combined with aerosolized antibiotics. In an adequately-powered study, Rattanaumpawan and colleagues found no difference in favorable clinical response between aerosolized colisthmethate and aerosolized saline placebo, when used with intravenous antibiotics at the discretion of the treating physician [14]. A small pilot study (*n* = 10) by Hallal et al. [9] found that 100 % of VAP patients treated with aerosolized tobramycin and intravenous β-lactam antibiotics had clinical resolution of VAP, compared to 60 % of those receiving combined intravenous tobramycin and β-lactam antibiotics; however, this result was not statistically significant difference due to low power. Niederman et al. [11] randomized subjects to an investigational form of amikacin (BAY41-6551) every 12 h, every 24 h or placebo every 12 h. The primary outcome in the study was the combination of tracheal aspirate amikacin maximum concentration ≥ 6400 μg/mL and ratio of area under aspirate concentration-time curve to minimum inhibitory concentration-time curve (0 –24 h) to minimum inhibitory concentration ≥ 100 on day 1. Fifty percent and 16.7 % of their patients achieved the primary endpoint in the every 12 h and every 24 h groups, respectively. Secondary outcomes such as clinical cure rates were not significantly different between the every 12 h, every 24 h and placebo groups, respectively (*p* = 0.467). However, a statistically significant difference in mean antibiotics per patient per day was seen between the three groups, 0.9 in the every 12 h, 1.3 in the every 24 h and 1.9 in the placebo groups, respectively (*p* = 0.02 between the groups). In the single study that compared aerosolized antibiotics alone to intravenous antibiotics, Lu et al [10] found a 21.4 % difference between nebulized and intravenous amikacin/ceftazidime with respect to successful treatment (70 vs 55 %; *p* = 0.33), a 50 % reduction in treatment failure (15 vs 30 %; *p* = 0.26), and no difference superinfection with other microorganisms (15 vs 15 %; *p* = 1.00) [10]. Overall, no studies demonstrated that AA were associated with poorer patient outcomes.

### Risk of bias assessment (Table 2)

The risk of bias varied across the six studies evaluated in this systematic review. All studies had low or unclear risk of selection bias. Three studies [10, 11, 14] had high risk of performance bias due to lack of blinding. Two studies [10, 11] had high risk of detection bias due to lack of blinding for those making the clinical cure assessments, while one study [14] had unclear risk of detection bias because they did not state who made the assessments of clinical cure. Two studies [11, 12] had high risk of attrition due to inability to conduct intention-to-treat analysis [11] or high termination rate [12]. One study [12] had high risk of reporting bias, as they did not report clinical outcomes for all patients. Finally, two studies [11, 12] had high risk of conflict of interest bias given that they were industry sponsored, while two additional studies [9, 13] did not discuss funding sources or other conflicts of interest. Overall, only two studies (Hallal [9], Palmer [13]) were assessed with low or unclear risk of bias across all domains, while Niederman [11] had a high risk of bias assessed across five of six domains.

## Discussion

Our systematic review found six articles that report that aerosolized antibiotics may improve clinical cure for VAP, particularly when combined with intravenous antibiotics. The strengths of the current systematic review include use of the Cochran Collaboration/PRISMA [7, 8] guidelines for reporting of systematic reviews and the inclusion of only randomized controlled trials. All included studies had a study arm that used either placebo aerosolized medications or only systemic antibiotics for comparison. Included studies used aerosolized antibiotics with a similar spectrum of antibacterial coverage. Finally, the majority of the studies included had high external validity in allowing treating clinicians’ discretion to determine use of systemic antibiotics and other intensive care measures.

Of the studies included, two reported statistically significant clinical improvement when both nebulized and intravenous antibiotics were delivered, compared to intravenous antibiotics alone [12, 13]. Four studies showed no statistical differences in clinical cure between patients who received IV or aerosolized antibiotics [10] or when nebulized antibiotics were added to intravenous antibiotics [9, 11, 14]. Overall, only three studies [12–14] had adequate power to detect a difference in clinical cure rate; while the three remaining studies were pilot studies [9, 10] or were powered for another clinical outcome [11]. Only one study used aerosolized antibiotics without systemic antibiotics; this study showed a clinically meaningful but non-statistically significant 21.4 % relative increase in successful treatment using aerosolized antibiotics [10].

One potential explanation for the disparate results involves the antibiotic nebulization technique. Achieving adequate treatment of any pulmonary infection via inhaled antibiotics requires delivery of sufficient antibiotics to the lungs. This involves adequate nebulization of the antibiotics into appropriate particle size for delivery in high concentrations. Previous research demonstrates that certain types of nebulizers (e.g. jet nebulizers) may be less efficient at antibiotic delivery than other methods (e.g ultrasonic or vibrating plate nebulizers) for patients on mechanical ventilation [15]. Only three of the studies [11–13] included used nebulizers with data that demonstrated that they had adequate antibiotic delivery to the airway or lung. Of the three papers using untested nebulizers, one used a non-specified vibrating plate nebulizer [10], another a Pari-Jet nebulizer [9] and the last did not specify the nebulizer type [14]. Of all six studies, the two studies that found positive results had appropriate nebulizers [12, 13]. Thus, differences seen in efficacy of inhaled antibiotics may be due to differential delivery of antibiotics to the target tissue.

This systematic review has several limitations. First, we excluded studies that were not randomized, controlled trials written in English. By excluding observational studies, such as cohort and case-control studies, we decreased the number of studies examined. This may have affected the external validity of this systematic review by missing studies showing significant changes that might affect the overall estimate of effect sizes. Second, the primary study outcome of the systematic review, clinical cure, was not defined uniformly in each of the included studies. Thus, there may be differential misclassification of the study outcome leading to information bias that might cause over- or underestimation of the effect size. While all were randomized, controlled trials, two [10, 14] were not blinded fully and relied on non-blinded evaluation of clinical cure and two [11, 12] were sponsored in part by pharmaceutical companies. Unblinded studies may introduce bias by allowing the outcome evaluators to misclassify study outcomes due to treatment groups. Fourth, in five of the six studies, all study participants were given intravenous antibiotics at the direction of the treating physician. Therefore, we cannot determine the efficacy of using solely aerosolized antibiotics, when compared to systemic antibiotics in the treatment of VAP or VAT. Lastly, publication bias may affect the current study’s conclusions, as we may have missed studies that met inclusion criteria but that were not published or studies presented in abstract form that were submitted to conferences but never published. However, that four out of six studies had null findings suggests that there was less publication bias specifically due to the absence of statistically significant results.

## Conclusion

This systematic review found insufficient evidence for the use of inhaled antibiotic therapy as primary or adjuvant treatment of VAP or VAT. Given the variations in study protocols, antibiotics studied and vague definition of clinical cure as an outcome measure, additional, adequately powered randomized-controlled trials with strict definitions of outcome assessments and use of previously validated nebulizer delivery methods for antibiotic administration are needed to assess the efficacy of inhaled antibiotic therapy for VAP and VAT.

## Additional file

Additional file 1: PubMed Electronic search method. (PDF 10 kb)

## Abbreviations

AA: aerosolized antibiotics
HAI: healthcare-associated infection
ICU: intensive care unit
LOS: length of stay
VAP: ventilator-associated pneumonia
VAT: ventilator-associated tracheobronchitis
