# Supplementary material for: The use of inhaled antibiotic therapy in the treatment of ventilator-associated pneumonia and tracheobronchitis: a systematic review
Source: BMC Pulm Med. 2016 Mar 8;16:40. doi: 10.1186/s12890-016-0202-8 (PMC4784295; doi:10.1186/s12890-016-0202-8)
Supplement: Additional file 1: — PubMed Electronic search method. (PDF 10 kb) [file 12890_2016_202_MOESM1_ESM.pdf]

Additional File 1. PubMed Electronic search method

Search terms included:
("Bronchitis"[Mesh] OR bronchitis OR "tracheobronchitis" OR "Pneumonia, Ventilator-Associated"[Mesh] OR Ventilator-associated Pneumonia OR VAP) AND (("Administration, Inhalation"[Mesh] OR "Nebulizers and Vaporizers"[Mesh] OR "Administration, Intranasal"[Mesh] OR aerosolized OR nebulized OR nebulizer OR vaporizer OR intranasal administration OR inhalation administration OR vaporized) AND ("Anti-Bacterial Agents"[Mesh] OR "Anti-Bacterial Agents" [Pharmacological Action] OR antibiotics OR anti-bacterial agents OR antibacterial agents OR Bacteriocidal Agents OR bacteriocides OR anti-mycobacterial agents OR 2,4-diacetylphloroglucinol OR 2-deoxystreptamine OR Acedapsone OR aconiazide OR actinonin OR actinorhodin OR Alamethicin OR albomycin OR Amdinocillin OR Amdinocillin Pivoxil OR amifloxacin OR Amikacin OR Aminosalicylic Acid OR Amoxicillin OR Amoxicillin-Potassium Clavulanate Combination OR amphomycin OR Amphotericin B OR Ampicillin OR amprenavir OR angustmycin A OR Anisomycin OR antibiotic 1233A OR Antimycin A OR antofloxacin OR apramycin OR Arsphenamine OR Aurodox OR avilamycin OR Azithromycin OR Azlocillin OR Aztreonam OR bacampicillin OR Bacitracin OR bacitracin zinc, neomycin sulfate, polymyxin B, drug combination OR bacitracin, cysteine, glycine, neomycin, threonine drug combination OR Bacteriocins OR balofloxacin OR Bambermycins OR bedaquiline OR bekanamycin OR benzathine benzylpenicillin, procaine benzylpenicillin, drug combination OR benzathine cloxacillin OR berythromycin OR beta-Lactams OR bialaphos OR bicozamycin OR blasticidin S OR Bongkrekic Acid OR bredinin OR Brefeldin A OR broadcillin OR Butirosin Sulfate OR cactinomycin OR Calcimycin OR Candicidin OR Capreomycin OR Carbenicillin OR carbenicillin indanyl OR Carfecillin OR Cefaclor OR Cefadroxil OR Cefamandole OR cefamandole nafate OR Cefatrizine OR cefazedone OR Cefazolin OR cefdinir OR cefditoren OR cefditoren pivoxil OR cefepime OR cefetamet OR cefetamet pivoxyl OR Cefixime OR Cefmenoxime OR Cefmetazole OR cefminox OR cefodizime OR Cefonicid OR Cefoperazone OR ceforanide OR cefoselis OR Cefotaxime OR Cefotetan OR Cefotiam OR Cefoxitin OR cefpimizole OR cefpiramide OR cefpirome OR cefpodoxime OR cefpodoxime proxetil OR cefprozil OR Cefsulodin OR Ceftazidime OR cefteram pivoxil OR ceftezole OR ceftibuten OR ceftiofur OR Ceftizoxime OR ceftobiprole OR ceftobiprole medocaril OR Ceftriaxone OR Cefuroxime OR cefuroxime axetil OR Cephacetrile OR Cephalexin OR Cephaloglycin OR Cephaloridine OR Cephalosporins OR Cephalothin OR Cephamycins OR Cephapirin OR Cephradine OR cethromycin OR chelerythrine OR Chloramphenicol OR chloramphenicol succinate OR chlorotetracycline, penicillin G, sulfamethazine drug combination OR chloroxine OR Chlortetracycline OR Ciprofloxacin OR ciprofloxacin, hydrocortisone drug combination OR Citrinin OR Clarithromycin OR Clavulanic Acid OR Clavulanic Acids OR clinafloxacin OR Clindamycin OR clindamycin palmitate OR clindamycin phosphate OR Clofazimine OR Cloxacillin OR Colistin OR Cyclacillin OR Cycloserine OR Dactinomycin OR dalbavancin OR dalfopristin OR Dapsone OR Daptomycin OR decamethoxine OR Demeclocycline OR desoxyfructo-serotonin OR dexamethasone, neomycin, polymyxin B drug combination OR Diarylquinolines OR Dibekacin OR Dicloxacillin OR Dihydrostreptomycin Sulfate OR Diketopiperazines OR dirithromycin OR Distamycins OR diucifon OR Doxycycline OR dynemicin A OR Echinomycin OR Edeine OR efrotomycin OR emiglitate OR Enoxacin OR Enviomycin OR epicillin OR ertapenem OR Erythromycin OR Erythromycin Estolate OR Erythromycin Ethylsuccinate OR erythromycin lactobionate OR erythromycin stearate OR essential 303 forte OR Ethambutol OR Ethionamide OR Filipin OR FK 565 OR florfenicol OR Floxacillin OR Fluoroquinolones OR forphenicinol OR Fosfomycin OR Framycetin OR fumagillin OR fusafungin OR Fusidic Acid OR gamithromycin OR garenoxacin OR gatifloxacin OR GE 2270 A OR gemifloxacin OR Gentamicins OR Gramicidin OR grepafloxacin OR herbimycin OR hydrocortisone, neomycin, polymyxin B drug combination OR Hygromycin B OR Imipenem OR immunomycin OR isepamicin OR Isoniazid OR isoniazid, pyrazinamide, rifampin drug combination OR Josamycin OR Kanamycin OR Kitasamycin OR KRM 1648 OR KT 5720 OR lactacystin OR Lactams OR lacticin 481 OR lactoferricin B OR Lasalocid OR Leucomycins OR Levofloxacin OR Lincomycin OR Lincosamides OR lomefloxacin OR loracarbef OR Lucensomycin OR lydiamycin A OR Lymecycline OR maduramicin OR maltotetraose OR manoalide OR manumycin OR marbofloxacin OR meclocycline OR Mepartricin OR meropenem OR Methacycline OR methampicillin OR Methicillin OR mevastatin OR Mezlocillin OR micronomicin OR midecamycin OR Mikamycin OR Minocycline OR Miocamycin OR mirincamycin OR mocimycin OR Moxalactam OR moxifloxacin OR muconomycin A OR Mupirocin OR Mycobacillin OR N-methyldeoxynojirimycin OR nadifloxacin OR Nafcillin OR Nalidixic Acid OR narasin OR Natamycin OR Nebacetin OR Nebramycin OR nebularine OR Neomycin OR Netilmicin OR Netropsin OR Nigericin OR Nisin OR nojirimycin OR Norfloxacin OR Novobiocin OR Nystatin OR Ofloxacin OR Oleandomycin OR Oligomycins OR ormetoprim, sulfadimethoxine drug combination OR Oxacillin OR oxetanocin OR Oxolinic Acid OR Oxytetracycline OR panipenem-betamipron OR Paromomycin

OR pazufloxacin OR pediocin PA-1 OR Pefloxacin OR Penicillanic Acid OR Penicillic Acid OR Penicillin G OR Penicillin G Benzathine OR Penicillin G Procaine OR Penicillin V OR penimepicycline OR phenethicillin OR phosphoramidon OR piericidin A OR Pipemidic Acid OR Piperacillin OR piperacillin-tazobactam combination product OR Pivampicillin OR Polymyxin B OR Polymyxins OR polyoxorim OR PR 39 OR Pristinamycin OR Prodigiosin OR propicillin OR Prothionamide OR prulifloxacin OR Pyrazinamide OR pyrazofurin OR quinupristin OR quinupristin-dalfopristin OR radezolid OR ramoplanin OR Ribostamycin OR Rifabutin OR rifamexil OR Rifampin OR Rifamycins OR rifapentine OR Ristocetin OR Rolitetracycline OR Roxarsone OR Roxithromycin OR Rutamycin OR saframycin A OR salinomycin OR sangivamycin OR Sirolimus OR Sisomicin OR sitafloxacin OR SM-4300 OR sodium thiosulfate OR sparfloxacin OR Spectinomycin OR Spiramycin OR squalamine OR staphylococcin OR stigmatellin OR Streptogramin A OR Streptogramin Group A OR Streptogramin Group B OR Streptogramins OR Streptomycin OR Streptovaricin OR Sulbactam OR Sulbenicillin OR sulfaguanol OR Sulfamerazine OR Sulfameter OR Sulfamethoxypyridazine OR sulfanilamide OR sultamicillin OR suncillin OR syringomycin OR Talampicillin OR Teicoplanin OR telavancin OR telithromycin OR temafloxacin OR temocillin OR tetarimycin A OR tetracenomycin C OR Tetracycline OR Thalidomide OR Thiamphenicol OR thienamycin OR Thienamycins OR Thioacetazone OR thiobenzamide OR thiocarlide OR thiolactomycin OR Thiostrepton OR thymopoietin III OR tiamulin OR Ticarcillin OR ticarcillin-clavulanic acid OR tigecycline OR tilmicosin OR Tobramycin OR tomaymycin OR triostin A OR Troleandomycin OR Tunicamycin OR Tylosin OR Tyrocidine OR Tyrothricin OR ubenimex OR ulifloxacin OR undecylprodigiosin OR Valinomycin OR Vancomycin OR VD 2085 OR Vernamycin B OR Viomycin OR Virginiamycin))
